# Statistical methods for the analysis of high-throughput metabolomics data

**DOI:** 10.5936/csbj.201301009

**Published:** 2013-03-22

**Authors:** Jörg Bartel, Jan Krumsiek, Fabian J. Theis

**Affiliations:** a Institute of Bioinformatics and Systems Biology, Helmholtz Zentrum München, Ingolstädter Landstr. 1, 85764 Neuherberg, Germany; b Department of Mathematics, Technische Universität München, Boltzmannstr. 3, 85747 Garching, Germany

## Abstract

Metabolomics is a relatively new high-throughput technology that aims at measuring all endogenous metabolites within a biological sample in an unbiased fashion. The resulting metabolic profiles may be regarded as functional signatures of the physiological state, and have been shown to comprise effects of genetic regulation as well as environmental factors. This potential to connect genotypic to phenotypic information promises new insights and biomarkers for different research fields, including biomedical and pharmaceutical research. In the statistical analysis of metabolomics data, many techniques from other omics fields can be reused. However recently, a number of tools specific for metabolomics data have been developed as well. The focus of this mini review will be on recent advancements in the analysis of metabolomics data especially by utilizing Gaussian graphical models and independent component analysis.

## Metabolomics in the field of biomedical research

With the advent of metabolomics, a new, important milestone in the endeavor to fully measure a biological system could be achieved. Metabolomics refers to the quantitative assessment of all metabolites (small molecules) within a biological system [1]. The analytical techniques predominantly used for the quantification are mass spectrometry (MS) and nuclear magnetic resonance (NMR) spectroscopy both having different strength and weaknesses [2–5]. There exist two main strategies for the quantification and identification of metabolites, the choice of which mainly depends on the experimental question to be answered. *Targeted metabolomics* is the method of choice in a hypothesis driven experiment, i.e. if the research focus lies on one or more particular metabolic pathways that are known to play a role in the examined biochemical setting. Only a predefined panel of metabolites is quantified, allowing for a precise snapshot of the desired physiological context. In contrast to that, *untargeted metabolomics* aims to measure ideally all endogenous metabolites contained in a biological sample providing a global and unbiased picture of a system's metabolism. However, the chemical identification and functional characterization of many yet unknown compounds measured in an untargeted metabolomics approach remains a substantial challenge [6]. Typically, compounds are identified by comparing the measured masses to those of known metabolites stored in databases such as HMDB [7], LipidMaps [8] and Metlin [9] besides others (see e.g. http://www.metabolomicssociety.org/database). We will not go into further details on the different data preprocessing steps here since they differ greatly between the particular analytical platforms available for measuring the metabolome. However, a comprehensive review dealing with this topic can be found in [10]. Applications of metabolomics can be found in a huge diversity of research fields, including environmental perturbations of biological systems, toxicology, disease diagnosis and biomarker identification. Biomarkers are measurable biological indicators that can be used for instance in clinical screenings to stratify patients according to the characteristics of their phenotype [11].

The suitability of metabolites as molecular biomarkers was demonstrated in several recent publications. Suhre et al. [12, 13] and Gieger et al. [14] showed that changes in the concentration levels of biochemically related metabolite pairs are often highly correlated with genetic variation in the general population. Specifically, they report that a SNP in the proximity of the coding regions of genes is frequently associated with variations in the concentration levels of metabolites which the protein processes or transports. Mohit et al. [15] examined the concentration changes of metabolites from NCI-60 cancer cells along with gene expression data. They reported a strong correlation between glycine consumption, the expression of glycine biosynthetic pathway related genes and the proliferation rate of cancer cells. Further successful applications were demonstrated both in nutritional challenge studies [16, 17] and in the investigation of molecular cell mechanisms [18, 19].

In the early days, biochemical approaches typically focused on a very limited amount of metabolites keeping the results manually interpretable by the researchers [20, 21]. However, being a very active field of research, metabolomics has made rapid progress nowadays allowing modern instrumentation to measure thousands of metabolites simultaneously. This growing complexity of high-throughput small molecule measurements now constitutes a substantial challenge to the researchers. The question that arises is how to derive biological meaningful results given thousands of chemically distinct metabolites measured in a specific experiment. In order to answer this question, robust statistical methods suitable to analyze and functionally interpret the complex interactions between the thousands of analytes are required.

The intention of this mini review is to give a coarse overview of the field of metabolomics and to briefly discuss the most commonly used statistical methodologies for the analysis of metabolomics data. Whenever possible, we provide the reader with references on two or more application examples as well as comprehensive articles or reviews dealing with theoretical aspects of the methodologies. Because of the broad application field of metabolomics and to keep a common focus we have chosen to mainly select human studies on disease diagnostics or biomarker identification as application examples. In the remainder of this mini review, two statistical concepts recently applied to high-throughput metabolomics data by our group will be especially emphasized: network modeling based on Gaussian graphical models (GGMs) and higher-order correlation analysis denoted as independent component analysis (ICA).

## Common statistical analysis techniques for metabolomics data

**Figure 1:**
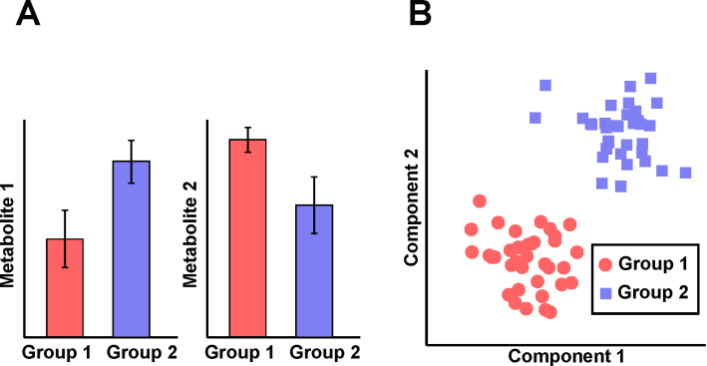
**Classical approaches to analyze metabolomics data. A)** Differences in the concentration level of single metabolites between two or more groups (e.g. t-test, ANOVA). **B)** Multivariate approaches like PCA and PLS model the relationships between metabolites and/or samples to detect group differences. Data points represent observations (samples).

Methodologies used to interpret high-throughput metabolomics data are mainly adapted from earlier emerged *omics* technologies, mostly originally developed for transcriptomics analysis. Classic analytical approaches aim to assess group-wise differences, either in a univariate i.e. parameter-by-parameter fashion (e.g. t-test, analysis of variance (ANOVA), see Figure 1A) or using multivariate techniques (e.g. MANOVA, ASCA, PCA, PLS, see Figure 1B). Univariate methodologies are frequently used to reduce a possibly large number of measured analytes to only those that show the strongest response under the investigated conditions. Examples for such univariate approaches are a two-way ANOVA to investigate medication-induced level changes of individual metabolites [22] or a Wilcoxon rank-sum test combined with ANOVA to delineate different cancer progression states ranging from benign prostate to the metastatic disease [23]. However, univariate methods fail to discriminate between groups if there are only minor differences on single molecule level, even if multi-molecule combinations would delineate them on a systems level.

Therefore, multivariate analysis methods seek to capture not only changes of single metabolites between different groups, but also to utilize the dependency structures between the individual molecules. Probably the most prominent multivariate analysis techniques applied in the field of metabolomics are principal component analysis (PCA), cluster analysis and partial least squares regression (PLS) including derivative methods.

PCA represents an *unsupervised* linear mixture model that attempts to explain the variance within a dataset by a smaller number of mutually decorrelated principal components (PCs) [5, 24]. In the case of metabolomics data, these PCs are vectors of metabolite contributions. All of the PCs are constructed such that they are pairwise orthogonal (decorrelated) to each other and ordered by the amount of variance they explain. PCA can be interpreted as linear mixture model, where the data matrix is factorized into two matrices: A score matrix, which contains the positions of the observations in a new, rotated coordinate system and a loading matrix, which contains the weights for the original variables to transform them into the scores. Because of its applicability in dimensionality reduction, data visualization, clustering and sample group discrimination (Figure 1B), PCA is often used as a starting point for data analysis, especially in a hypothesis free, exploratory experimental setup. Some applications amongst many in the field of metabolomics are the analysis of urine metabolomics in kidney cancer diagnostics [25] or urine and serum metabolites in Parkinson's disease [26] and diabetes [27].

A related multivariate method recently developed by Smilde et al. [28] is ANOVA-simultaneous component analysis (ASCA), a combination of ANOVA and PCA methodologies. ASCA is particularly suited for the analysis of datasets with a complex underlying experimental design, consisting of many simultaneously measured covariates. It thereby allows to directly relate variation in the data to the different design factors. Further theoretical aspects about ASCA as well as application examples on metabolomics data, e.g. the effect of oral rinse on human saliva metabolic profiles can be found in [29–31].

Cluster analysis represents another unsupervised multivariate technique suitable for the analysis of metabolomics data with self-organizing map (SOM) [32, 33], hierarchical cluster analysis (HCA) [34, 35] and k-means clustering [36, 37] being the most prominent representatives. In general, clustering methods group and visualize samples according to intrinsic similarities in their measurements, irrespective of sample groupings. Notably, some authors point out general issues of clustering approaches, like error propagation, difficult interpretability and poor reproducibility of the identified clusters [38, 39]. We will not go into detail here but refer the interested reader to a comprehensive review on clustering methods [40].

PLS regression, which belongs to the class of *supervised* linear mixture models, attempts to find an optimal decomposition of the predictor dataset given a matrix of responses. The general idea behind supervised methods is to unravel inherent patterns, e.g. distinct metabolite profiles that are strongly associated with the predefined response structure. For example, PLS-DA (discriminant analysis, i.e. with a categorical response), relates the data matrix (e.g. multivariate metabolite data) to the response vector (containing the sample class affiliations, e.g. case-control) by a linear regression model. The detailed procedure is elaborated elsewhere [41, 42]. PLS-DA is usually used for classification purposes either to infer the variables that maximize the discrimination between predefined sample groups or even to predict class affiliations of unclassified samples based on a calibration set of known class distributions. PLS-DA was applied for instance to discriminate healthy individuals from Crohn's disease patients on metabolomics data [43] or in the diagnosis of different types of cancer [25, 44, 45].

A recent extension to the PLS repository is the orthogonal-PLS (OPLS) [46] method. The main difference to classical PLS analysis is to split up the data variation into the variance of interest which is related to the response and an orthogonal (noise) part which is unrelated to the response. This leads to a simplified interpretability of the resulting components allowing to additionally asses within- and between-group variance [46–48]. OPLS has drawn attention in metabolomics research recently with a broad variety of classification applications including molecular epidemiology [35], alternative medicine [49] and the monitoring of kidney transplant patients [50].

A general limitation of supervised methods is the risk of overfitting [51] which means an incorporation of noise into the statistical model, e.g. caused by excessive learning on a training dataset. Yet there exists a number of validation techniques like cross validation [52] or bootstrapping [53] to overcome this issue but are not further discussed here.

It has to be noted that the selection of multivariate statistical methods discussed here is far from being complete and that a detailed critical review of the methods is beyond the scope of this mini review. Hence, the interested reader is referred to the pertinent literature [38, 42, 54–57]. In the following, we will focus on Gaussian graphical models and Independent Component Analysis, which have recently been applied to metabolomics data by our group.

## Gaussian graphical models

Cellular components, like metabolites, are members of strongly intertwined biological pathways and thus show a high degree of interactivity. A way to systematically model and intuitively interpret such interdependencies is the depiction as a graph or network [58]. This approach has become popular and widely used over the last decade. Networks typically consist of nodes, usually representing molecules (genes, proteins, metabolites), while links between the nodes depict their interactions. In a metabolic network, a node represents a metabolite and a link corresponds to a metabolic interaction (e.g. a biochemical reaction). Currently, several publicly available metabolic databases exist that focus on global reconstructions of metabolic pathways, like KEGG [59], human Recon 1 [60] and EHMN [61]. The biological networks contained in such databases can be used to guide statistical analysis in a functional manner. The concept of a network-guided analysis was applied to, for instance, classify different cancer subtypes by the identification of condition-specific activity patterns of PPI-networks or metabolic pathways [62, 63] or even for the inference of an individualized therapy [64].

Obviously, pathway databases are far from being complete, a fact which introduces bias to pathway-based analyses urging the need for new complementing strategies. One alternative, unbiased approach is to reconstruct pathway networks directly from the data. While this approach has been attempted and was of limited success in the context of regulatory networks in particular in mammalian cells [65], the biochemical nature of metabolite reactions has shown to be better suited for such a reconstruction [66].

In order to reconstruct metabolic pathways directly from the data, statistical methods exploit the naturally occurring biological variation in the abundance of metabolites between biological replicates. Such variation in metabolite concentrations could occur either due to intrinsic fluctuations (e.g. in temperature or pH) [67] or due to extrinsic factors (e.g. changes in enzyme levels caused by different regulatory states) [68] in the system. It is important to note here that these variations in metabolite concentration often occur in a concerted way, as such reflecting the wiring of the underlying metabolic network. Common methods for the reconstruction of pathways from high-throughput data are based on Bayesian networks [69] or correlation-based measures [67]. Bayesian networks are probabilistic graphical models which depict random variables (e.g. gene expression levels or metabolite concentrations) as nodes and their conditional dependencies as directed edges. Prominent application examples are gene networks inferred from gene expression data [70, 71]. However, amongst other issues, Bayesian networks can only reconstruct acyclic graphs, while real biological networks are well-known to contain cycles and feedback loops [72, 73].

A quite direct and simple possibility to circumvent this limitation are pairwise correlation methods, where two nodes are connected if their respective correlation lies above a certain threshold [74]. Although their usefulness has been shown in several applications [75–77], standard correlation-based methods lack the ability to discriminate between direct and indirect associations: a high correlation between two metabolites could be mediated by one or more confounding variables which are the actual cause for the observed correlation [78, 79].

**Figure 2:**
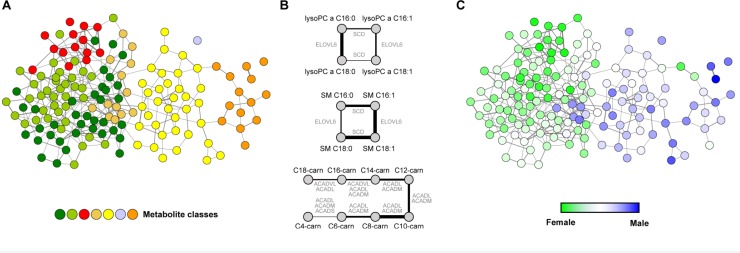
**Gaussian graphical models applied to metabolomics data. A)** Network representation of a Gaussian graphical model. Each node corresponds to a metabolite, whereas each edge represents a significant partial correlation. **B)** Reconstructed subgraphs correspond to known biological reactions. Line widths indicate partial correlations strength; Edges are labeled with enzymes that supposedly account for the observed correlation. We observe effects of fatty acid desaturation and elongation in phospholipids, as well as beta-oxidation signatures for acyl carnitines. lysoPC = lyso phosphatidylcholine, SM = sphingomyelin, carn = carnitine. **C)** The same GGM from A colored with gender-specific effects from a differential statistical analysis. In part adapted from [58].

Gaussian graphical models (GGMs), which are based on so-called *partial* correlation coefficients, eliminate indirect interactions by conditioning each pairwise association between two variables against all remaining variables. A GGM is an undirected graph, where each node corresponds to a random variable and an edge between two nodes is drawn if the variables are conditionally dependent given all other variables [80] (Figure 2A). GGMs have attracted some attention in the field of transcriptomics analysis [81, 82] and, more recently, also in the analysis of metabolomics data [83, 84]. However, the calculation of full-order partial correlations usually requires a higher number of samples than variables [78], a demand often not met by current experimental designs. Several approaches addressed this issue, suggesting alternative estimation algorithms utilizing low-order partial correlations [81], bootstrap resampling [78] or shrinkage estimation [85].

Our group recently applied GGMs to targeted high-throughput metabolomics data [66]. In this work, we systematically demonstrated that GGMs are capable of recovering metabolic reactions solely from human blood plasma metabolomics data. In a first step, we generated *in silico* metabolomics data from different computer-simulated reaction systems, and found a clear advantage of GGMs over standard correlation networks. While correlation networks typically fail in recovering the true underlying network structure, GGMs perform well for most scenarios. The application to real metabolomics data from a population cohort and subsequent comparison to existing metabolic pathway databases revealed that high partial correlation coefficients generally coincide with known metabolic reactions (Figure 2B). In addition to that, several novel candidates for pathway interactions could be identified. Further applications to other datasets not only confirmed these findings, but also revealed the potential of GGMs in the identification of biomarkers. For example, in a study on gender inequalities, a Gaussian graphical model helped to discover sex-specific differences on the metabolite level [86] (Figure 2C). Moreover, Jourdan et al. [87] established a link between fat-free mass index and several blood serum metabolites by utilizing GGMs. In addition, several authors suggest methods to extend the undirected partial correlation information to the inference of directional networks. These include approaches based on partial variance [88], directed partial correlations [89] or the d-separation principle [90].

As already mentioned earlier, the chemical identification of yet unknown metabolites in mass spectrometry remains a key issue. Often, these unknown compounds cannot be assigned because current metabolic libraries lack entries or at least details on a non-negligible number of metabolites. We recently addressed this issue and successfully identified several compounds by utilizing GGMs in combination with genomic data [91], further hinting at the broad range of possible applications of Gaussian graphical models for functional metabolomics.

## Bayesian independent component analysis

Despite their powerful capabilities in the analysis of multivariate data, a drawback of methods like PLS-DA, PCA and GGMs is their limitation to second-order statistical dependencies (i.e. covariance) between the variables. Higher order dependencies, possibly deriving from non-linear metabolic processes, are inherently neglected by these classical statistical approaches. The linearity of associations between measured entities is an approximation which is only correct for precisely normally distributed data. We have shown in our previous studies that even after log-transformation (thus assuming log-normality), notable deviations from the normal distribution can be detected for a large fraction of metabolites [66, 91]. Note that a simple way to deal with non-normally distributed data is to use rank correlation (Spearman) or mutual information [92].

Independent component analysis (ICA) is a method that is able to capture higher order dependencies by extending the concept of regular correlation to statistical dependence. ICA has recently attracted attention in the field of biomedical research [93]. First applications were reported in the neurobiological field, including electroencephalographic (EEG) [94] and functional magnetic resonance imaging (fMRI) [95, 96]. Even in molecular biology, ICA found a use in the classification of cancer types [97, 98] and in the examination of the cell proliferation process [99, 100] from transcriptomics data. More recently, ICA was also discovered to be a promising method for metabolomics analysis, for instance, when dealing with plant toxins [101] or for the investigation of starch metabolism in *Arabidopsis thaliana* [102] and the development of colitis in mice [103].

**Figure 3:**
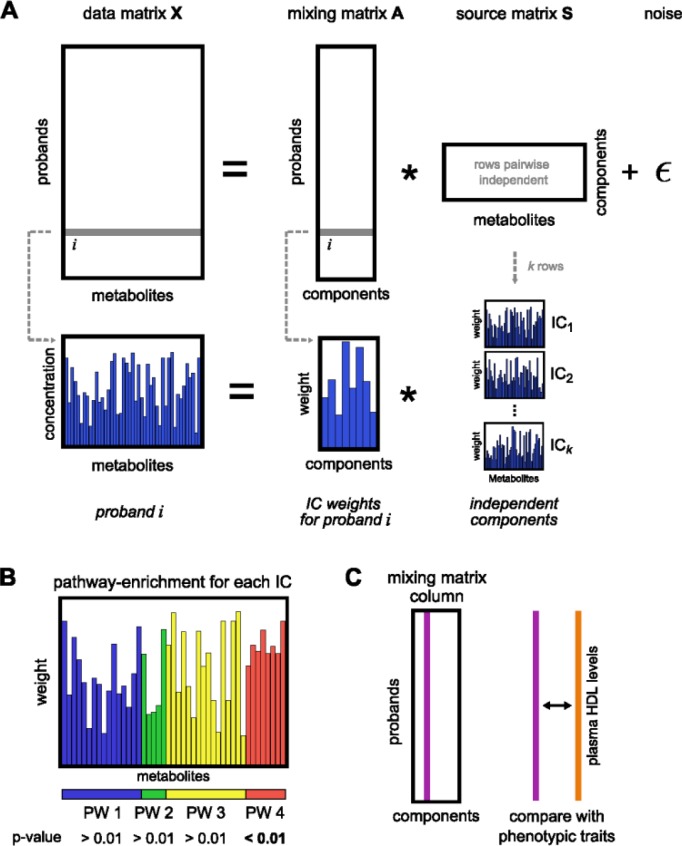
**Concept of Bayesian independent component analysis. A)** The data matrix of metabolite concentrations X is factorized into a mixing matrix A (containing contributions for each component in each proband) and a source matrix S (of statistically independent components, ICs). **B)** Functionally, we check for enriched metabolic pathways in each of the ICs to determine whether this statistical construct contains biological information. **C)** The mixing matrix values for each proband can be correlated with other traits, e.g. plasma HDL levels. Reprinted with permission from [76]. Copyright (2012) American Chemical Society.

While conceptually related to PCA, the main mathematical difference between ICA and PCA lies in the relation between the determined components. As mentioned above, ICA extends the decorrelation concept from PCA to statistical independence, a stronger condition if the data is non-Gaussian. For this purpose, ICA decomposes the data matrix of measured metabolomics profiles into *k* statistically independent components (ICs). A biological rationale behind this is the mixture of different biological processes (e.g. pathways) each of which contributes to a certain extent to the overall metabolic profile of the living system. Hence, metabolomics measurements represent a combination of these distinct metabolic processes that we seek to disentangle. Mathematically, a factorization of the data matrix into a mixing matrix A and a source matrix S has to be found (Figure 3A). These two matrices allow for different interpretations: each row in S can be seen as a particular metabolic process mixing up to the overall metabolic profile, whereas A indicates how strong each process is activated in a study sample.

A major challenge for ICA is the determination of a reasonable number of components *k*. There have been several suggestions from the ICA community on how to select k, mostly based on heuristics [104]. Moreover, classical ICA does not allow for the incorporation of prior information. Both issues can be tackled by employing a Bayesian ICA approach. Additionally, in such a framework, the Bayesian information criterion (BIC) [105] can be used to obtain the optimal number of independent components.

We recently applied a Bayesian mean-field ICA method [106] to metabolomics data, setting a nonnegativity constraint for both matrices as prior [107]. We argued that nonnegative contributions are biologically more reasonable than arbitrary values, since the concentration of metabolites cannot be negative and also the activity of a biological process should be positive or zero. The metabolomics data was derived from the German KORA F4 cohort and consists of 1764 blood serum samples and 218 measured metabolites covering various pathways (see [13] for details). By applying ICA to the metabolomics data and comparison to *k*-means clustering and a standard PCA, we were able to show that ICA outperforms the other methods in terms of a biologically more sound decomposition of the data. More precisely, the independent components showed a strong enrichment of distinct metabolic pathways (see Figure 3B and [107] for details) as opposed to for instance PCA, which showed an inconsistent distribution of the metabolites. On a side note, a similar study of ICA on gene expression data also reported a stronger biological enrichment as opposed to *k*-means and PCA [108].

Moreover, correlating IC's to blood plasma HDL (high-density lipoprotein) levels revealed a strong association with one particular IC (Figure 3C). HDL is a specific class of lipoproteins which transports lipophilic molecules like cholesterol and triglycerides in the blood plasma. HDL has long been known to be associated with a variety of biological processes and is therefore of particularly high clinical interest [109, 110]. An inspection of the independent component revealed a high contribution of branched-chain amino acids which possibly indicates a yet unknown association between branched-chain amino acids and HDL blood plasma levels.

## Conclusion

A wide spectrum of analysis techniques for metabolomics data have already been proposed, including various standard analysis methods such as t-test and ANOVA, as well as more sophisticated methodologies. In general, we argue that the combination of different methods, thereby combining their complementing features, represents a promising approach allowing the researcher to extract the best-possible amount of information from an experiment. Furthermore, future experimental designs have to be adjusted to the capabilities of existing methodologies, for instance keeping in mind adequate sample sizes. Despite that, all of the studies discussed in this mini review highlighted the potential of existing methods for analyzing metabolomics data ranging from the reconstruction of pathway reactions to the identification of disease biomarkers and the delineation of chemical identities. These studies further increased our understanding not only of cellular and physiological biochemistry but also of the functional mechanisms underlying the onset and progression of particular diseases. Indeed, changes in the abundance of metabolites in response to pathophysiological states are a direct consequence of the underlying biological processes (gene function and enzyme activity) including environmental factors, which renders them a promising link between genotype and phenotype [111].

**Figure 4:**
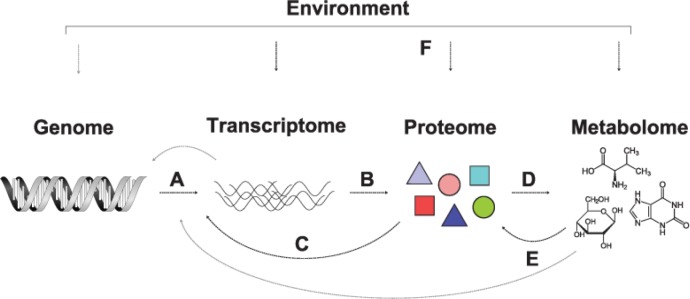
**Flow of biological information**. Genomic information is transcribed into RNAs **(A)**, which thereafter are translated into proteins **(B)**. Proteins act in the regulation of transcription (e.g. as transcription factors, **C**) or directly on metabolite levels as enzymes or transporters **(D)**. Metabolites, in turn, can regulate the activity of proteins for instance as ligands or via protein modifications **(E)**. All organizational levels are affected by environmental factors like diet, lifestyle or mutagenic exposure **(F)**.

However, given the complexity of biological systems - which are controlled by different levels of biological regulation, all highly dynamically interacting with each other (Figure 4) - it is unlikely that biomarkers from a single layer (e.g. metabolites) bear the ability to explain an individual's phenotype. Each organizational level, like the transcriptome or metabolome, yields distinct information about physiological and cellular processes. Therefore, data from multiple molecular entities have to be analyzed in a multivariate and integrative manner to be able to capture not only the subtle changes on single molecule level, but also changes of the interconnectivity between the cellular components. A major challenge that is already addressed by several groups is the development of adequate methodologies capable of integrating measurements from multiple levels of biological organization. First progress has already been made in this field with successful integration of multiple *omics* datasets in different research areas, including the analysis of *E. coli* stress response [112], or in human biology with a comprehensive analysis of metabolomics, transcriptomics and genomics data of a population cohort [113]. Moreover, in a tremendous effort, genomic, transcriptomic, proteomic and metabolomic profiles were measured and integratively analyzed from a single individual for a period of 14 month with the ultimate goal of an individually tailored treatment [114]. As the quality and resolution of metabolomics measurement techniques proceeds, an integrative analysis of different high-throughput datasets on single cell level will become possible [115]. We expect that the increasing quality of the available data will not only lead to the development of new statistical methods but also to an improved performance of existing analysis techniques eventually providing even deeper insights into the complete picture of an organism's biology.
